# Propolis extract nanoparticles alleviate diabetes-induced reproductive dysfunction in male rats: antidiabetic, antioxidant, and steroidogenesis modulatory role

**DOI:** 10.1038/s41598-024-81949-z

**Published:** 2024-12-23

**Authors:** Abram B. Emil, Neven H. Hassan, Sally Ibrahim, Eman I. Hassanen, Zienab E. Eldin, Sara E. Ali

**Affiliations:** 1 Department of Physiology, Faculty of Veterinary Medicine, Cairo University, Giza, 12211 Egypt; 2 Department of Animal Reproduction and AI, Veterinary Research Institute, National Research Centre, Dokki, Giza, 12622 Egypt; 3 Department of Pathology, Faculty of Veterinary Medicine, Cairo University, Giza, 12211 Egypt; 4 Materials Science and Nanotechnology Department, Faculty of Postgraduate Studies for Advanced Science (PSAS), Beni-Suef, 62511 Egypt

**Keywords:** Propolis extract nanoparticles, Diabetes, Oxidative stress, Steroidogenesis, Male rats, Chemical biology, Drug discovery, Physiology, Systems biology, Endocrinology, Medical research

## Abstract

Diabetes can affect male fertility via oxidative stress and endocrine system disruption. Nanomedicine based on natural products is employed to address diabetes complications. The current study aims to investigate the potential beneficial effect of propolis extract nanoparticles against diabetes-induced testicular damage in male rats. Sixty male rats were randomly allocated to six groups (*n* = 10). The first group served as a control group. The second and third received propolis extract (Pr) and propolis extract nanoparticles (PrNPs). The fourth group is the diabetic group that received streptozotocin (STZ) (55 mg kg/bwt) single-dose i/p. The fifth and sixth groups are diabetic rats treated with Pr and PrNPs. Both Pr and PrNPs were received at a dose (100 mg/kg bwt) orally. After 60 days, animals were euthanized, then pancreatic and testicular tissues were collected for redox status evaluation, gene expression analysis, and histopathological examination. Also, hormonal analysis (Insulin, total testosterone, and luteinizing hormone (LH) ) along with semen quality evaluation were done. Results showed that the induction of diabetes led to testicular and pancreatic redox status deterioration showing a reduction in reduced glutathione (GSH) as well as elevation of malondialdehyde (MDA), and nitric oxide (NO) levels. Also, relative transcript levels of testicular *cytochrome P450 family 11 subfamily A member 1 (CYP11A1)*,* 3β-Hydroxysteroid dehydrogenase (HSD-3β)*,* and nuclear factor (erythroid-derived 2)-like 2 (NFE2L2)* were significantly down-regulated, While the advanced glycation end-product receptor *(AGER)* relative gene expression was significantly upregulated. Furthermore, hormonal and semen analysis disturbances were observed. Upon treatment with Pr and PrNPs,  a marked upregulation of testicular gene expression of *CYP11A1*,* HSD-3β*,* and NFE2L2* as well as a downregulation of *AGER,* was observed. Hormones and semen analysis were improved. In addition, the testicular and pancreatic redox status was enhanced. Results were confirmed via histopathological investigations. PrNPs outperformed Pr in terms of steroidogenesis pathway improvement, testicular antioxidant defense mechanism augmentation, and prospective antidiabetic activity.

## Introduction

Diabetes mellitus (DM) is a complex and diverse condition that can be generally classified into two categories: type 1 diabetes (T1DM) and type 2 diabetes (T2DM). T1DM is defined by a severe or total lack of insulin, while T2DM is identified by insulin resistance and inadequate compensatory insulin production^1^. Consequently, diabetes disrupts the metabolism of carbohydrates, fats, and proteins, which leads to long-term complications affecting multiple organs^2^. Disturbances in the male reproductive system are a common complication of DM, often resulting in sexual dysfunction due to the disruption of glucose metabolism, which is essential for spermatogenesis^3,4^. A decline in the steroidogenic capacity in individuals with diabetes has been previously reported^5^. That is linked to lower plasma testosterone levels, due to the downregulation of androgen-related genes, including hydroxysteroid dehydrogenases (HSDs) and cytochrome P450 (CYPs)^6^. Also, diabetes was reported to encourage the formation of advanced glycation end products (AGEs) that can exacerbate reproductive functions^7^.

However, the exact molecular mechanism is still controversial. It has been proposed that DM leads to various reproductive complications due to a combination of endocrine dysfunction, oxidative stress, testicular tissue damage, and poor sperm quality^8,9^.

Thus, exploring potential therapeutic strategies to mitigate fertility-related complications caused by DM is essential. Antioxidants are thought to have several beneficial impacts on DM as they help combat complications associated with reactive oxygen species (ROS)^10^. Natural antioxidants play a vital role in managing DM and its complications, particularly in developing countries^11^. They have the potential to postpone the onset of diabetic complications, rectify metabolic irregularities, and protect against damage caused by oxidative stress induced by ROS^12^.

Propolis, also referred to as bee glue, is a sticky resinous substance created by honeybees through the combination of their waxes and plant resins^13^. Historically, propolis extracts have been a staple in folk medicine due to their potent antioxidant properties and diverse biological effects including antibacterial, anticancer, anti-inflammatory, liver protective, and immunomodulatory effects^14,15^. Propolis is considered an intricate substance that comprises over 300 components, encompassing phenolic aldehydes, polyphenols, sesquiterpene quinones, coumarins, steroids, amino acids, and inorganic compounds^16^. Notably, among these components, phenolic compounds, especially flavonoids, are primarily responsible for propolis’s beneficial effects due to their radical scavenging antioxidant activity^17^. Furthermore, propolis combats free radicals by enhancing the enzymatic function of antioxidants and overall antioxidant levels, reducing lipid peroxidation, and inhibiting the formation of superoxide anions and nitric oxide^17^. Such properties are particularly valuable for protecting the reproductive system from harmful side effects, thus preventing impairment to semen quality, testosterone levels, and testicular function^18^. Research has indicated that propolis can increase sperm motility seminal plasma enzymes, reproductive organ weight, and testosterone levels, in addition to lowering free radical levels^19,20^. However, despite its numerous health benefits, The utilization of propolis is constrained by various challenges, including low water solubility, limited bioavailability, susceptibility to oxidation, and significant biotransformation restrictions^21,22^.

In recent years, the intersection of nanotechnology with natural therapeutic agents has opened new avenues in medical research, especially in managing chronic conditions like diabetes^23,24^. This focused study aims to explore the innovative impact of propolis extract nanoparticles and their potential therapeutic effects on male fertility issues associated with diabetes aiming to enhance their bioavailability, solubility, and therapeutic efficacy, overcoming the limitations faced by conventional forms^25^. Studies have indicated that nano propolis exhibits superior antioxidant, antibacterial, anti-inflammatory, and antifungal activities^26,27^, which could be beneficial in mitigating diabetes-induced damage to male reproductive organs. Despite the promising outlook, research in this domain, especially concerning the ameliorative effects of nano propolis on male fertility in diabetic contexts, is limited. This study aims to shed light on the current trends, potential molecular mechanisms, and future insights of propolis nanoparticles as a novel therapeutic adjunct therapy for diabetes-related male infertility.

## Methods

### Chemicals

Egyptian bee Propolis was obtained from the Agriculture Research Center.Tween-80 was acquired from (Thermo Fisher Scientific USA). Streptozotocin (purity 98%) (CAS No. 18883-66-4) and citrate buffer solution (10 mmol/L, pH4.5) were purchased from Sigma-Aldrich Co.

### Preparation of propolis aqueous extract

Propolis powder (10 gm) was combined with 100 milliliters of deionized water and left at ambient temperature for six hours. The combination was filtered using a syringe filter and kept ready at − 4 °C.

### Preparation and characterization of propolis extract nanoemulsion

The study utilized a low-energy thermal spontaneous emulsification method to create propolis Nanoemulsions, modifying existing techniques^28^. Pure propolis powder was dissolved in double-deionized distilled water with Tween 80 as a surfactant, then stirred and sonicated before filtration through a 200 nm filter. Characterization included X-ray diffraction (XRD) and Fourier transform infrared spectroscopy (FTIR) for structural analysis, field emission scanning electron microscopy (FESEM), and high-resolution transmission electron microscopy (HRTEM) for morphology. Dynamic light scattering (DLS) measured particle size and polydispersity, while zeta potential assessed nanoparticle stability.

### In vitro cytotoxicity study

#### The MTT (3-[4,5-dimethylthiazol-2-yl]-2,5 diphenyl tetrazolium bromide) assay

Vero cells were cultured in 96-well plates and incubated until nearly confluent. Control wells received fresh media, while experimental wells were exposed to different concentrations of propolis and nano propolis (0–1000 µg/mL). After 24 h, cells were washed and incubated with MTT solution, followed by dimethyl sulfoxide DMSO to dissolve formazan crystals. Optical density at 570 nm was measured to assess cell viability. Cell viability percentage was determined by dividing treated cells’ optical density (OD) by the OD of untreated cells and multiplying by 100.

### High-performance liquid chromatography (HPLC) analysis

This analysis utilized an Agilent 1260 system with a Zorbax Eclipse Plus C8 column. The mobile phase comprises water (A) and acetonitrile with 0.05% trifluoroacetic acid (B). The gradient began at 82% A, adjusted to 75% A from 1 to 11 min to 60% A from 11 to 18 min, and back to 82% A until 24 min. Detection was at 280 nm, with a 5 µl sample injection and the column temperature held at 40 °C.

### Headspace solid-phase microextraction gas chromatography mass spectrometry (HS-SPME-GC-MS) analysis

After conditioning, HS-SPME was conducted using PDMS and DVB-CAR-PDMS fibers. Initially, 2 g of raw propolis were placed in a vial and equilibrated at 75 °C, followed by exposure of the fiber for 20 min. Analytes were desorbed in the GC injector at 230 °C, and fibers were reconditioned for 5 min before reuse. To extract the essential oil from propolis, it was distilled with water, dehydrated, and stored at + 4 °C. The essential oil was then prepared for GC-MS analysis. The chromatographic analysis utilized an HP-5 MS column with the oven temperature ranging from 40 °C to 280 °C at 3 °C/min. HS-SPME-GC-MS employed splitless injection, while HD-GC-MS used a 1:100 split ratio. Helium was the carrier gas at 0.7 ml/min, and injector, transfer line, and ion-source temperatures were set to 230 °C, 280 °C, and 230 °C, respectively. MS detection employed electron ionization at 70 eV. Retention indices were calculated using a hydrocarbon mixture, and compound identification was based on comparing retention times and MS patterns with reference standards and the NIST database. The quantities were expressed as percentage peak areas^29^.

### Animals and experimental design

Sixty male Sprague Dawley rats, weighing between 200 and 240 g and aged 8–10 weeks from VACSERA, Egypt, were housed at the Faculty of Veterinary Medicine, Cairo University, where they underwent a two-week acclimation period. The rats were kept in standard polypropylene cages throughout the study under controlled environmental conditions: temperature maintained at (22 ± 3) °C and humidity at (55 ± 5%). They were provided ad libitum access to food and water. All experimental procedures were performed according to the Guide for the Care and Use of Laboratory Animals (NIH), the Animal Research: Reporting of in vivo Experiments (ARRIVE) guidelines, and approved by the Institutional Animal Care and Use Committee (IACUC) of the Faculty of Veterinary Medicine, Cairo University, following the Animal Use Protocol (Vet CU 25122023856).

#### Induction of diabetes

The rats underwent an overnight fast and received a single i/p injection of freshly prepared streptozotocin at a dose of 55 mg/kg body weight, liquefied in citrate-buffered saline (CBS) kept at a cold temperature (0.1 M; pH 4.5)^30^. To prevent hypoglycemic shock and death resulting from extensive and rapid destruction of β-cells and subsequent large-scale insulin release, the rats were given 1 ml/kg of a 5% glucose solution continuously for the next 24 h^31^. Three days after the streptozotocin injection, blood samples were collected from the tail vein for fasting blood glucose measurement using a glucometer (Accu-Check, Roche Diagnostics, Pvt. Ltd.). Rats with glucose levels exceeding 250 mg/dL were regarded as diabetic animals^32^.

Following the induction of diabetes, the rats were distributed randomly into six groups, each containing 10 animals as follows:

Group 1: The control group was injected (i/p) with CBS (0.1 M; pH 4.5) and received normal saline orally.

Groups 2 and 3: Normoglycemic rats were orally administered 100 mg/kg of propolis extract (Pr) and propolis extract nanoparticles (PrNPs), respectively.

Group 4: The diabetic group received a single (i/p) injection of streptozotocin (STZ) at a dose of 55 mg/kg body weight on the first day and received normal saline orally.

Groups 5 and 6: Diabetic rats were orally administered 100 mg/kg of Pr and PrNPs, respectively.

The experiment was extended for 60 days, and all oral interventions were administered day after day. The STZ dosage (55 mg/kg) was established based on prior studies for the induction of type 1 diabetes^33–35^. Whereas, 100 mg/kg of propolis was found to exhibit potential antioxidant activity^35,36^ .

### Sampling

Blood samples were collected after the experiment using orbital sinus puncture technique. Samples were centrifuged at 4000 rpm for 15 min to separate plasma (collected with fluoride as an anticoagulant) and serum (collected without anticoagulant). Following this, all animals were euthanized by intraperitoneal injection of ketamine 90 mg/xylazine 10 mg, and semen was retrieved via epididymal dissection for semen analysis. Furthermore, the testis and pancreas were extracted from all animals and washed with normal saline. Tissue samples were separated into two parts: one part was preserved at -80 °C for gene expression analysis and oxidative stress assessment. The other part was fixed in 10% neutral buffered formalin for histopathological examinations.

### Evaluation of blood glucose level

Fasting plasma glucose (FPG) levels were measured using a glucose kit purchased from the Spectrum Company in Egypt, with CAT. No (250 001).

### Oxidative stress indices

Pancreatic and testicular tissue homogenate were utilized to assess oxidative stress markers. To prepare the tissue homogenates, the tissues were homogenized in 10 mL of cold buffer per gram of tissue and then centrifuged at 5000 rpm for 15 min at 4 °C. The supernatant was collected for further analysis. For MDA assessment, a 50 mM potassium phosphate buffer (pH 7.5) was used. The buffer for GSH consisted of 50 mM potassium phosphate (pH 7.5) with 1 mM EDTA, while a 50 mM phosphate buffer (pH 7.4) was used for NO.

Kits for malondialdehyde (MDA), reduced glutathione (GSH), and nitric oxide (NO) were purchased from Biodiagnostic Company in Egypt (CAT. No MD 25 29, GR 25 11, TA 25 13, NO 25 33 respectively).

#### Glutathione reduced (GSH) assay

The approach relies on the reduction of 5,5’ dithiobis (2-nitrobenzoic acid) (DTNB) with glutathione (GSH) to create a yellow molecule. The reduced chromogen is directly proportional to GSH content, and its absorbance at 405 nm was determined^37^.

#### Malondialdehyde (MDA) assay

Thiobarbituric acid (TBA) reacts with malondialdehyde (MDA) in the acidic medium at 95 °C for 30 min to produce the thiobarbituric acid reactive product; the absorbance of the ensuing pink product can be measured at 534 nm. MDA followed the specified protocols^38^,

#### Nitiric oxide (NO) assay

In the acid medium and in the presence of nitrite the formed nitrous acid diazotizes sulphanilamide and the product is coupled with N-(1-naphthyl) ethylenediamine. The resulting azo dye has a bright reddish-purple color which can be measured at 540 nm^39^.

### Hormonal analysis

Serum samples were used to quantify total testosterone, luteinizing hormone (LH), and insulin levels. Total testosterone levels were assessed using an ELISA kit from Gamma Trade (CAT: EELR005). ELISA assay kits from ELK Biotechnology were used to measure LH (CAT: ELK2367) and insulin (CAT: ELK2370) levels.

### Evaluation of testicular weight and semen analysis

Semen analysis was carried out using the technique previously discussed^40,41^. The freshly collected sperm samples were used to detect sperm cell concentration, motility, viability, and abnormalities. Furthermore, a precision weighing apparatus was utilized to determine the weight of the testis.

### Gene expression analysis (RT-qPCR)

Total RNA was extracted from testicular tissues using the miRNeasy mini kit (Cat. No. 217004; QIAGEN) as per the manufacturer’s instructions. Genomic DNA contamination was eliminated with on-column DNA digestion using the RNase-free DNase kit. The concentration and purity of RNA were assessed using Nano-drop 2000/c, and RNA integrity was confirmed by 2% agarose gel electrophoresis. Subsequently, the RNA was utilized for cDNA synthesis employing the Xpert cDNA Synthesis Supermix (5x) (Grisp, Portugal, #GK86.0100). For cDNA synthesis, a 20 µl reaction mixture containing 10 ng RNA, 4 µl Xpert cDNA Synthesis Supermix (5x), and 10 µl RNase-free water in an RNase-free microtube was prepared. The thermocycler conditions included 37 °C for 20 min, 60 °C for 10 min, 95 °C for 1 min, followed by holding at 4 °C and immediately chilling on ice. The synthesized cDNA was validated using a GAPDH primer in a PCR reaction and stored at − 20 °C. Gene-specific primers for *AGER, NFE212, CYP11A1*, and *HSD-3β* - (Table 1) were designed using Primer3 software (http://bioinfo.ut.ee/primer3-0.4.0/ )^42^.The transcript levels in testicular tissues of all groups of rats were quantified using the Xpert Fast SYBR (uni) 2X Master mix (Grisp, Portugal, #GE20.0100) and the Stratagene Mx 3000P instrument (Agilent Technologies, USA). Melting curve analysis was conducted to confirm the specificity of amplification. GAPDH expression levels served as internal controls for normalization, and their consistency across different experimental groups was validated using NormFinder software^43^.

**Table 1:** The primer sets used in the study.

Gene name	Sequence 5′–3′	Accession no	Amplicon
*GADPH*	F: TTGACCTCAACTACATGGTCTA R: CCAGTAGACTCCACGACATACT	XM_039107008.1	187
*AGER*	F: GAAGCTAGAATGGAAACTGAAC R: GTAGTTGGACTTGACCTCCTT	NM_053336.2	196
*NFE212*	F: CAGTTACAACTGGATGAAGAG R: AAGAAACCTCATGGTCATCTAC	XM_039105941.1	194
*CYP11A1*	F: CCAGAACTTCTACTGGGACTTA R: CTCCTGTACCTTCAAGTTGTGT	NM_012584.2	205
*HSD-3β*	F: CCTTAAGACTCCCATTCATCT R: TAGTAGAACTGTCCTTGGATGC	NM_012584.2	214

### Histopathological investigation

The collected samples were fixed in 10% neutral buffered formalin followed by a series of dehydration and clearance using ascending grades of alcohol and xylene. They were embedded in paraffin wax and sliced into 4.5 μm thick sections. All sections were stained by hematoxylin and eosin (H&E) and inspected under an Olympus BX43 light microscope to record any alterations. Afterward, images were taken at different magnification power (100–400 x) using Olympus camera DP21 attached to Cell Sense dimension software^44^.

Histological lesion scoring of pancreases was done by measuring the diameter of islets of Langerhans which reflect the degree of extent of diabetes in diverse groups. Whereas testicular changes were quantitatively scored by counting the spermatogenic cells in fifty seminiferous tubules (ST)/group following the Johnsen scoring system^45^. Ten-point graded scale was used as^10^ means homogenous ST lined by well organized, stratified germinal cells and intact Sertoli cells (SC), whereas^1^ means empty ST. The grading was done by a pathologist who blinded the treatment to avoid bias.

### Statistical analysis

The results were presented as means ± standard error. Statistical analysis involved conducting a one-way analysis of variance (ANOVA) followed by the Tukey post-hoc test using SPSS statistical analysis software (Version 20) to determine significant differences between groups. A p-value of less than 0.05 was considered statistically significant.

## Results

### PrNPs characterization

#### X-ray diffraction characteristics (XRD)

**Fig. 1:**
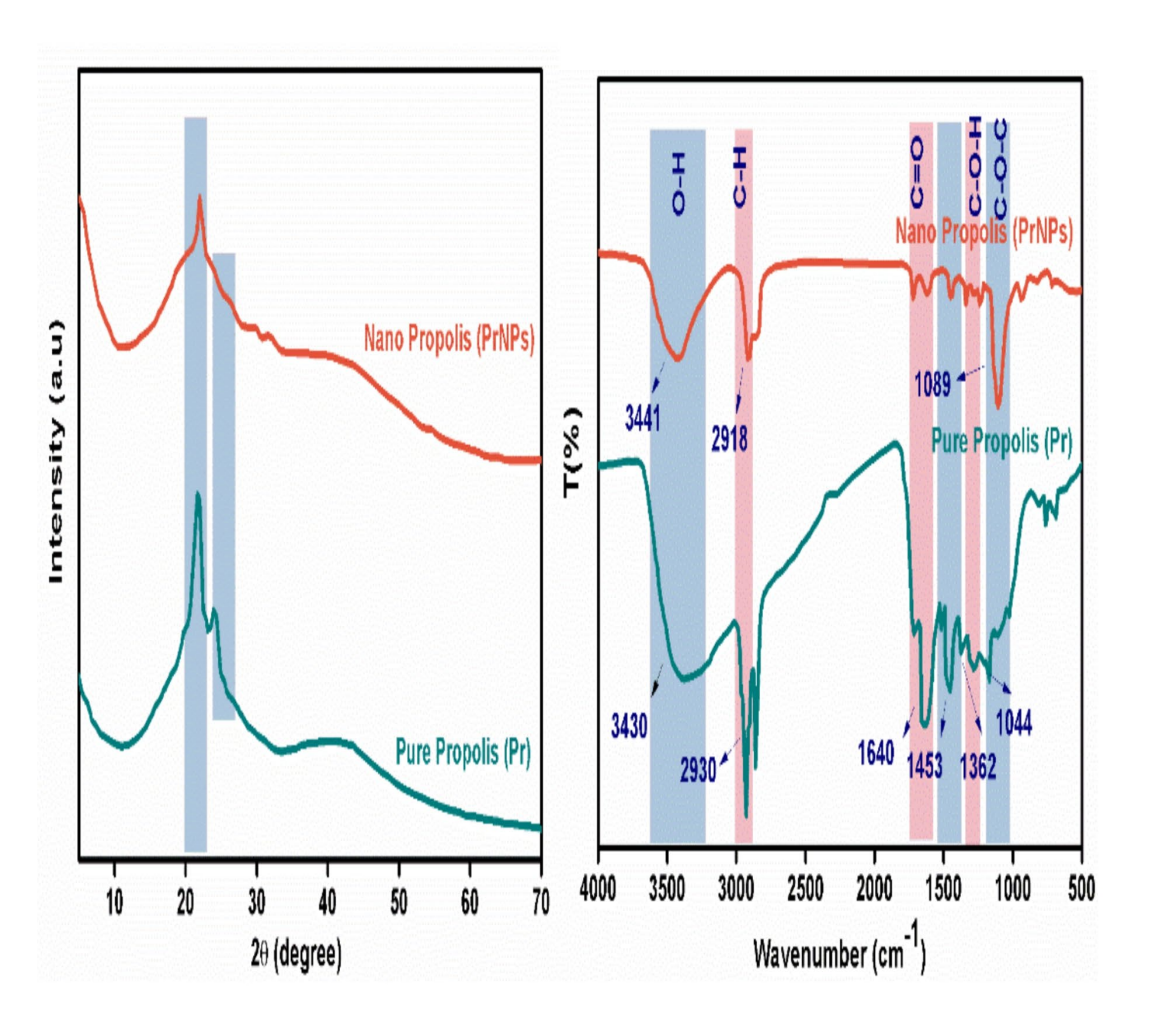
(**A**) XRD, and (**B**) IR spectra of Pure propolis (Pr) and Nanopropolis (PrNPs).

As depicted in Fig. 1A, the XRD pattern of Pure propolis exhibited characteristic, faint, narrow peaks at around 21.4° and 23.8°, suggesting the presence of a very low degree of crystalline material. The broad background region spanning from the beginning of the spectra to 70° indicates the highly amorphous nature of the product. Similar propolis diffractograms with peaks in this region have been reported previously. The intensity in nanopropolis decreased and shifted to 22.11°.

#### IR spectral studies

FTIR analysis was performed to examine structural differences between nano propolis extract and propolis extract. The FTIR spectrum of propolis (Fig. 1B) showed a peak at 3430 cm^−1^, indicating O-H stretching vibrations of phenolic compounds involved in hydrogen bonding. Additional peaks were observed at 1640, 1543, and 1453 cm^− 1^, corresponding to C=O stretching vibrations of flavonoids and lipids present in propolis. Peaks at 1362 and 1266 cm^− 1^ were attributed to C-O-H stretching vibrations, while the peak at 1158 cm^− 1^ indicated alkene C=C bonds. Specifically, a peak at 1044 cm^− 1^ was noted for the stretching vibration of aromatic ether C-O-C bonds. The FTIR analysis results revealed new bonds formed due to the conversion of propolis extract into nano-propolis compounds. The FTIR data showed that propolis nanoparticles have additional bonds compared to pure propolis extract, resulting from the nanosization process.

#### Surface morphology (SEM and TEM study)

**Fig. 2:**
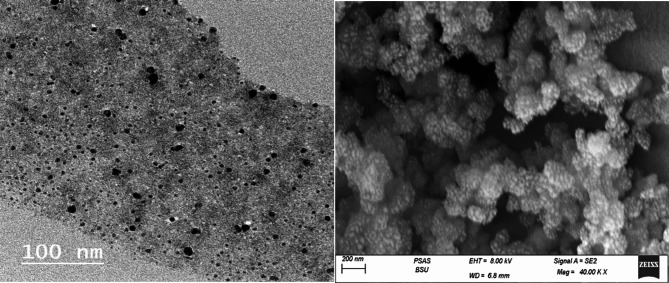
(**A**) TEM, (**B**) SEM photograph of Nanopropolis (PrNPs).

Surface structure and morphology analysis of the prepared propolis extract nanoparticles (PrNPs) utilized SEM and TEM techniques. As depicted in Fig. 2A, B, SEM imaging revealed that the nanoparticles had nearly spherical shapes with irregularly agglomerated particles. Through the TEM technique, the TEM images of PrNPs demonstrated agglomerate nearly spherical nanoparticles with sizes ranging between 18 and 42 nm.

#### Hydrodynamic size, PDI, and ζ-potential

**Fig. 3:**
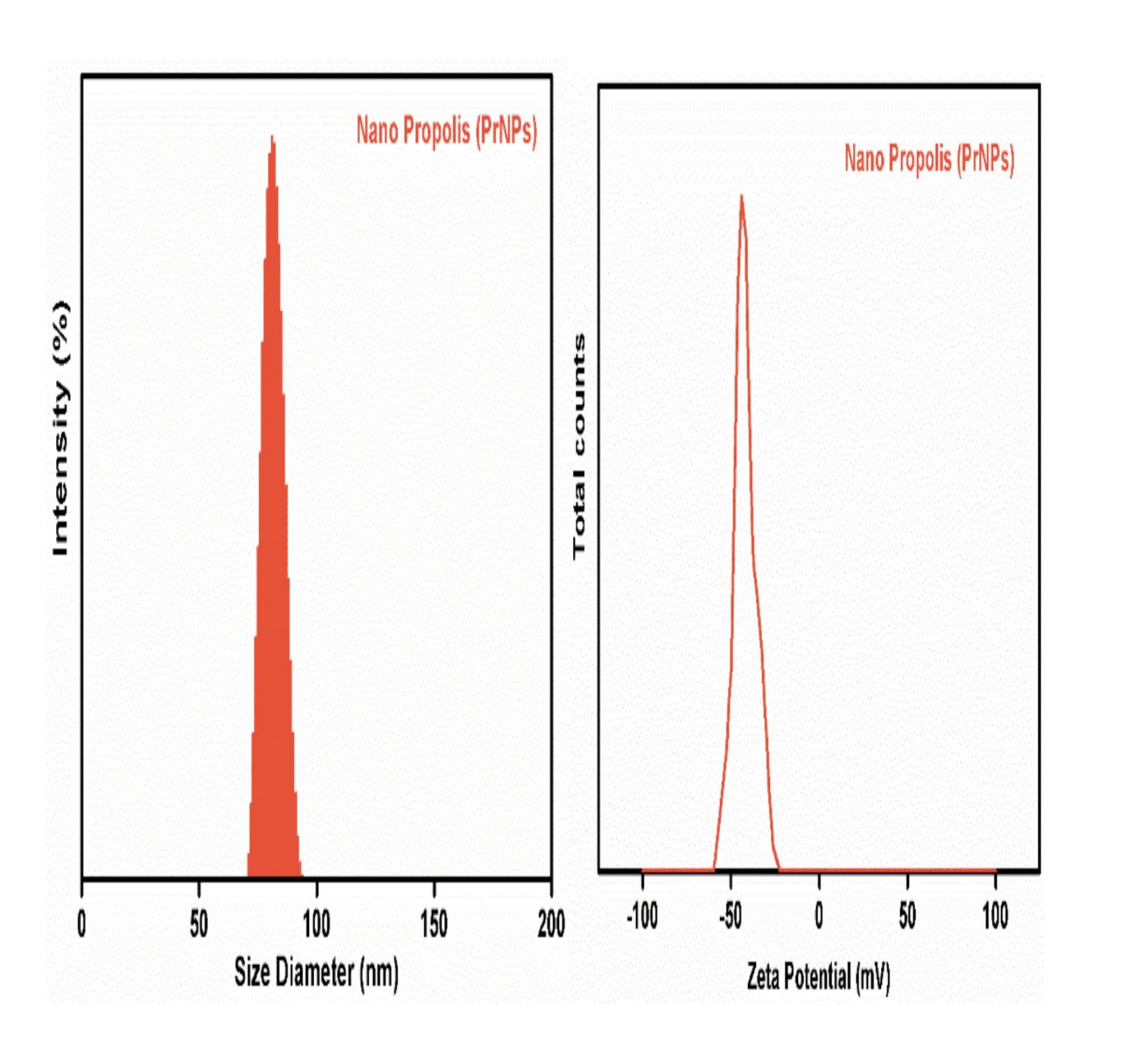
(**A**) Particle size analysis, (**B**) Zeta potential of nanopropolis (PrNPs),

To assess the physical characteristics and stability of the prepared propolis extract nanoparticles (PrNPs), measurements of particle size and zeta potential were conducted. DLS was employed to determine the hydrodynamic size distribution of the PrNPs, depicted in Fig. 3A,B. The measured size was 82 ± 7.2 nm with a polydispersity index (PDI) of 0.25 ± 0.08, which is below 0.7, indicating good stability of the PrNPs. The DLS size measurement closely matched TEM observations, showing minimal variation in nanoparticle size between the two techniques. In terms of particle stability, the Zeta potential was determined to be -40 ± 2 mV, confirming the stability of the PrNPs.

### In vitro cytotoxicity study

**Fig. 4:**
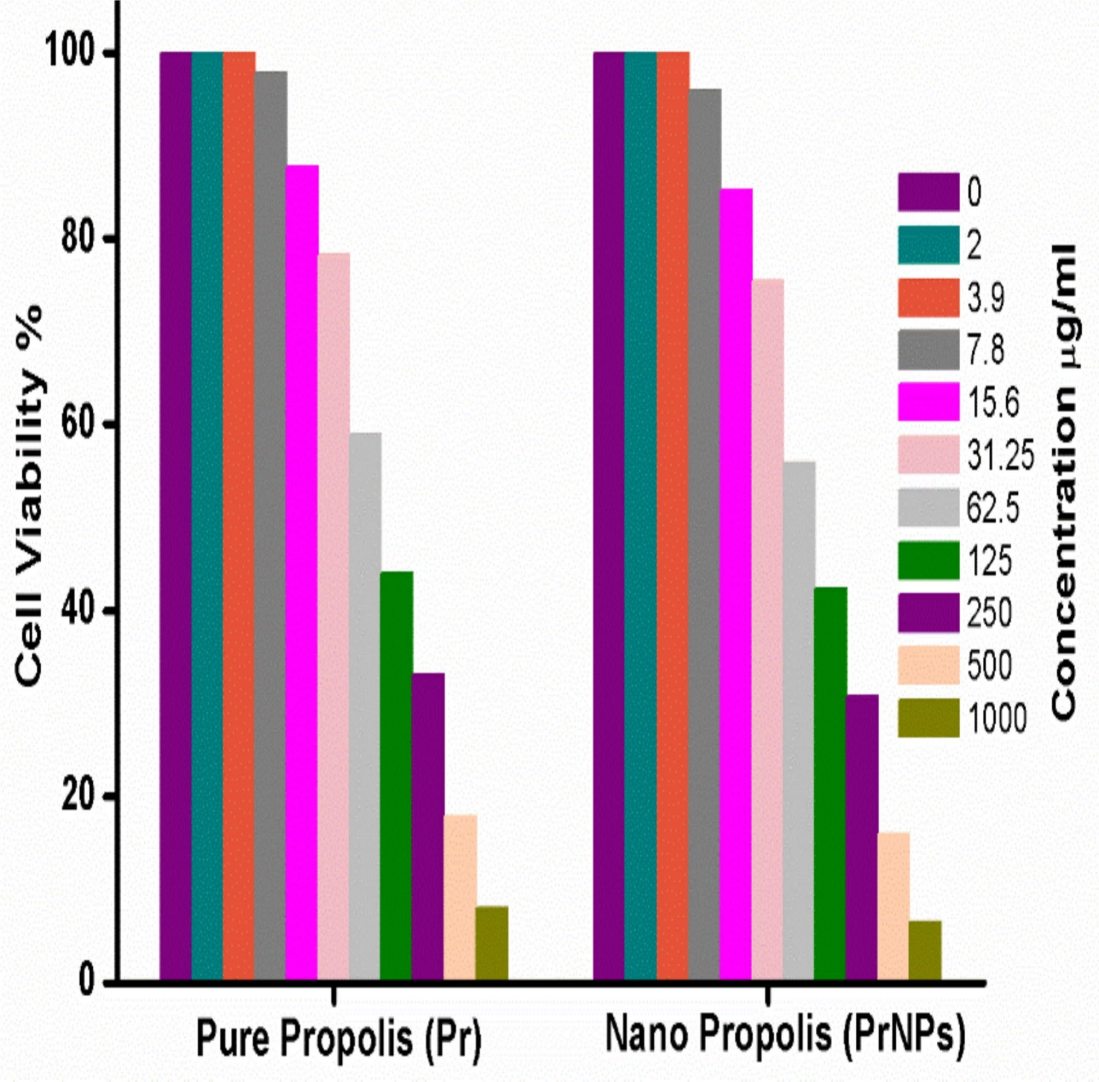
Cell viability percentage after incubation with Pure propolis (Pr) and Nanopropolis (PrNPs) for 24 h.

Figure 4 displayed the cell viability percentages of propolis extract (Pr) and propolis extract nanoparticles (PrNPs), revealing that both low and high doses of Pr and PrNPs have moderate cytotoxic effects on Vero cells. Furthermore, at the highest dose, Pr and PrNPs exhibited cell viabilities of approximately 98% and 97%, respectively. These results indicate the safety and biocompatibility of PrNPs at low doses for potential in vivo applications.

### High-performance liquid chromatography (HPLC) analysis

This study revealed significant amounts of gallic acid (59.778 µg/g), chlorogenic acid (27.362 µg/g), syringic acid (15.014 µg/g), and rosmarinic acid (10.422 µg/g) in propolis. Since these compounds are well-established antioxidants, their presence suggests that propolis likely possess potent antioxidant capabilities (Table 2) (*p* ≤ 0.05).

**Table 2:** High-performance liquid chromatography (HPLC) results of propolis extract.

Component name	Conc. (µg/ml)	Conc. (µg/g)
Gallic acid	2.99 ± 0.01	59.778 ± 1.23
Chlorogenic acid	1.37 ± 0.03	27.362 ± 1.15
Catechin	0.44 ± 0.2	8.876 ± 0.32
Methyl gallate	0.52 ± 0.02	10.367 ± 0.05
Coffeic acid	0.38 ± 0.01	7.504 ± 0.45
Syringic acid	0.75 ± 0.32	15.014 ± 0.58
Rutin	0.00	0.000
Ellagic acid	0.15 ± 0.01	2.940 ± 0.22
Coumaric acid	0.03 ± 0.02	0.612 ± 0.01
Vanillin	0.00	0.000
Ferulic acid	0.10 ± 0.01	1.953 ± 0.02
Naringenin	0.18 ± 0.01	3.607 ± 0.09
Rosmarinic acid	0.52 ± 0.3	10.422 ± 0.48
Daidzein	0.11 ± 0.01	2.275 ± 0.07
Querectin	0.98 ± 0.21	19.542 ± 0.02
Cinnamic acid	0.07 ± 0.03	1.385 ± 0.04
Kaempferol	0.16 ± 0.01	3.202 ± 0.06

Each value is expressed as mean ± standard error (*n* = 3; *P* ≤ 0.05).

### Headspace solid-phase microextraction gas chromatography mass spectrometry (HS-SPME-GC-MS) analysis

This study identified furfural (19.49%), dimethyl sulfide (6.02%), palmitic acid (5.1%), and nonanal (4.99%) as major components of propolis. Interestingly, these compounds are responsible for the characteristic odor associated with propolis (Table 3) (*p* ≤  0.05).

**Table 3:** Volatile compounds identified in raw propolis samples.

Peak	Component name	RT
1	Acetone	1.599 ± 0.15
2	Dimethyl sulfide	1.656 ± 0.12
3	Methyl formate	1.845 ± 0.31
4	Pentyl allyl ether	2.159 ± 0.16
5	2-Propenoic acid, hexyl ester	2.371 ± 0.01
6	2-Ethylfuran	2.491 ± 0.14
7	Hexanal	3.607 ± 0.15
8	Furfural	4.174 ± 0.01
9	(E)-2-Hexenal	4.517 ± 0.05
10	5-Methylfuran-2(3 H)-one	4.843 ± 0.16
11	3,5-Dimethyl-1-hexene	6.783 ± 0.12
12	Benzaldehyde	6.92 ± 0.05
13	Hexanoic acid	7.47 ± 0.09
14	2-Pentylfuran	7.658 ± 0.14
15	trans-2-(2-Pentenyl)furan	7.922 ± 0.03
16	Octanal	7.956 ± 0.22
17	2,4-Heptadien-1-al	8.185 ± 0.02
18	p-Cymene	8.562 ± 0.04
19	.(2E)-3-Propyl-2,4-pentadien-1-ol	8.929 ± 0.12
20	Benzeneacetaldehyde	9.1 ± 0.11
21	Decamethylcyclopentasiloxane	9.186 ± 0.01
22	1,5,5-Trimethyl-6-methylene-1-cyclohexene	9.489 ± 0.02
23	3,5-Octadien-2-one	9.833 ± 0.14
24	Nonanal	10.737 ± 0.06
25	2-(trans)-6-(cis)-Nonadienal	12.127 ± 0.01
26	Octanoic acid	12.768 ± 0.04
27	Decanal	13.575 ± 0.14
28	Nonanoic acid	15.395 ± 0.09
29	Dodecamethylcyclohexasiloxane	16.327 ± 0.17
30	2,6,10-Trimethyltetradecane	23.577 ± 0.12
31	Decanoic acid, hexadecyl ester	24.453 ± 0.01
32	Palmitic acid	31.164 ± 0.05
33	Phthalic acid, butyl tetradecyl ester	31.222 ± 0.07

Each value is expressed as mean ± standard error ( *n* = 3; *P* ≤ 0.05).

### Glucose level

Table 4 revealed a statistically significant rise in blood glucose levels among the diabetic group compared to the control group (*p* ≤ 0.05). A transformative effect was observed in both treated groups receiving either Pr or PrNPs, showing a remarkable improvement in blood glucose levels within the diabetic group. Notably, the diabetic + PrNPs group exhibited a significant enhancement in blood glucose levels compared to the Pr-treated diabetic group.

**Table 4:** The blood glucose levels in different experimental groups.

Group parameter	Glucose levels (mg/dl)
Control	94 ± 2.26^a^
Pr	90.8 ± 2.95^a^
PrNPs	86.1 ± 7.03^a^
Diabetic	528.6 ± 6.05^d^
Diabetic + Pr	416 ± 7.26^c^
Diabetic + PrNPs	310 ± 4.65^b^

Each value is expressed as mean ± standard error (*n* = 6 rats/group). This means having different letters in the same column is significantly different at *P* ≤ 0.05.

### Oxidative stress indices

**Fig. 5:**
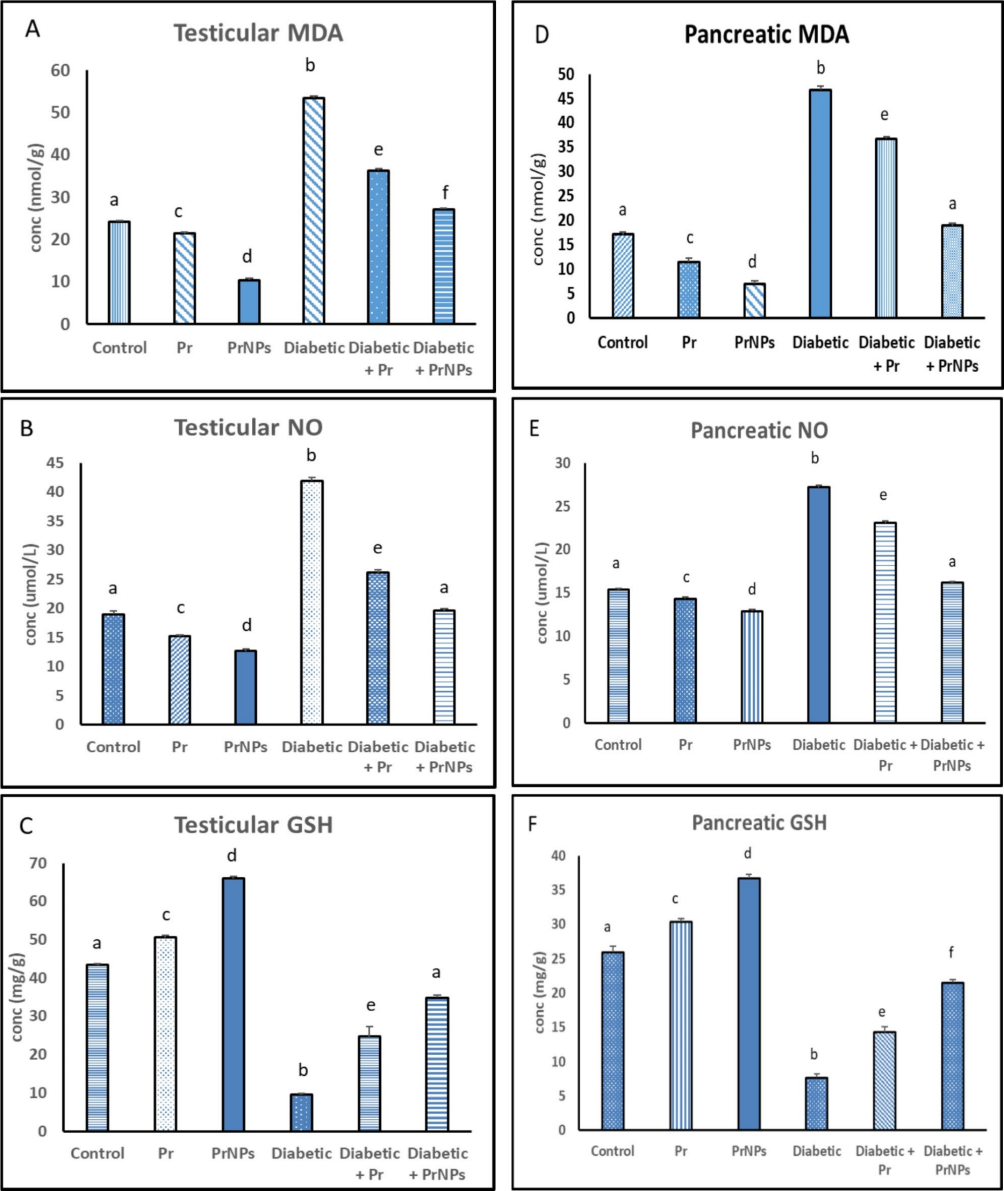
The testicular and pancreatic oxidative stress parameters in different experimental groups. Each value is expressed as Mean ± standard error (*n* = 6 rats/group). Means having different letters are significantly different at (*P* ≤ 0.05).

Figure 5 showed a significant rise in MDA and NO concentrations in the testicular and pancreatic tissues of the diabetic rats, accompanied by a corresponding decline in GSH levels comparable to the control group (*p* ≤ 0.05). Furthermore, Diabetic groups treated with Pr and PrNPs showed a significant decrease in MDA and NO levels, as well as a significant increase in GSH levels within both testicular and pancreatic tissues (*p* ≤ 0.05) compared to the untreated diabetic rats. Notably, the diabetic + PrNPs group exhibited a significant enhancement in testicular and pancreatic tissue levels of MDA, NO, and GSH comparable to the Pr-treated diabetic group. Moreover, in the pancreatic tissue of the diabetic + PrNPs group, levels of MDA, and NO, showed a remarkable restoration compared to the control level. Similarly, the testicular tissue of the diabetic + PrNPs group exhibited GSH and NO levels like those observed in the control group (*p* ≤ 0.05).

### Hormonal analysis

Table 5 showed a notable decrease in serum luteinizing hormone (LH), testosterone, and insulin levels in the diabetic rats comparable to the control rats (*p* ≤ 0.05). However, both Pr and PrNPs interventions led to a significant enhancement in those hormonal levels compared to the untreated diabetic group. Interestingly, PrNP therapy within the diabetic group yielded statistically significant improvement relative to the diabetic group that received Pr only (*p* ≤ 0.05). Furthermore, testosterone levels in the diabetic + PrNPs group showed no significant difference comparable to the control rats (*p* ≤ 0.05).

**Table 5:** Hormonal analysis in different experimental groups.

Group parameter	LH (pg/mL)	Testosterone (ng/mL)	Insulin (pg/mL)
Control	49.98 ± 0.15^d^	15.21 ± 0.21^c, d^	335.92 ± 5.40^d^
Pr	51.73 ± 0.1^e, d^	16.12 ± 0.12^e, d^	413.25 ± 3.37^e^
PrNPs	53.87 ± 0.29^e^	16.65 ± 0.18^d^	465 ± 5.43^f^
Diabetic	19.79 ± 1.29^a^	8.48 ± 0.55^a^	66.45 ± 7.13^a^
Diabetic + Pr	36.55 ± 0.49^b^	12.49 ± 0.17 ^b^	172.74 ± 4.29^b^
Diabetic + PrNPs	42.55 ± 0.59^c^	14.09 ± 0.16 ^c^	278.33 ± 5.47^c^

Each value is expressed as mean ± standard error (*n* = 6 rats/group). This means having different letters in the same column is significantly different at *P* ≤ 0.05.

### Evaluation of testicular weight and semen analysis

The findings of this study revealed a significant decrease in testicular weight and a compromised semen quality characterized by a significant reduction in viability, motility, and count, along with a significant increase in sperm abnormalities in the diabetic rats compared to the control group (*p* ≤ 0.05). However, both diabetic groups treated with either Pr or PrNPs showed significant improvements in semen analysis and restoration of testicular weight compared to the diabetic group (*p* ≤ 0.05). The diabetic group treated with PrNPs exhibited a statistically significant improvement surpassing that seen in the Pr-treated group (*p* ≤ 0.05). Notably, no statistically considerable difference was observed among testicular mass and sperm abnormalities in the diabetic rats treated with PrNPs and the control group (*p* ≤ 0.05) (Table 6).

**Table 6:** Markers of testicular function in different experimental groups.

Group parameter	Motility%	Viability%	Defect %	Concentration (*10^6^/ml)	Testes weight (g)
Control	80.5 ± 0.99^d^	58 ± 0.96^d^	54.16 ± 1.19^c^	76.35 ± 0.74^d^	1.40 ± .02^c^
Pr	84.6 ± 0.42^e^	65.41 ± 1.2^e^	41.83 ± 1.01^b^	82.81 ± 0.8^e^	1.49 ± 0.01^d^
PrNPs	89.8 ± 0.6^f^	75.41 ± 1.01^f^	25.50 ± 1.28^a^	90.9 ± 1.09^f^	1.52 ± .028^d^
Diabetic	23.16 ± 0.79^a^	20.3 ± 1.7^a^	93.16 ± 1.19^d^	46.91 ± 0.8^a^	0.30 ± 0.01^a^
Diabetic + Pr	53.3 ± 0.84^b^	38.5 ± 0.71^b^	62.83 ± 1.35^e^	62.58 ± 0.56^b^	0.95 ± 0.01^b^
Diabetic + PrNPs	65.5 ± 0.67^c^	45.73 ± 0.92^c^	54.50 ± 1.05^c^	69 ± 0.82^c^	1.34 ± .011^c^

Each value is expressed as mean ± standard error (*n* = 6 rats/group). This means having different letters in the same column is significantly different at *P* ≤ 0.05.

### Gene expression analysis

**Fig. 6:**
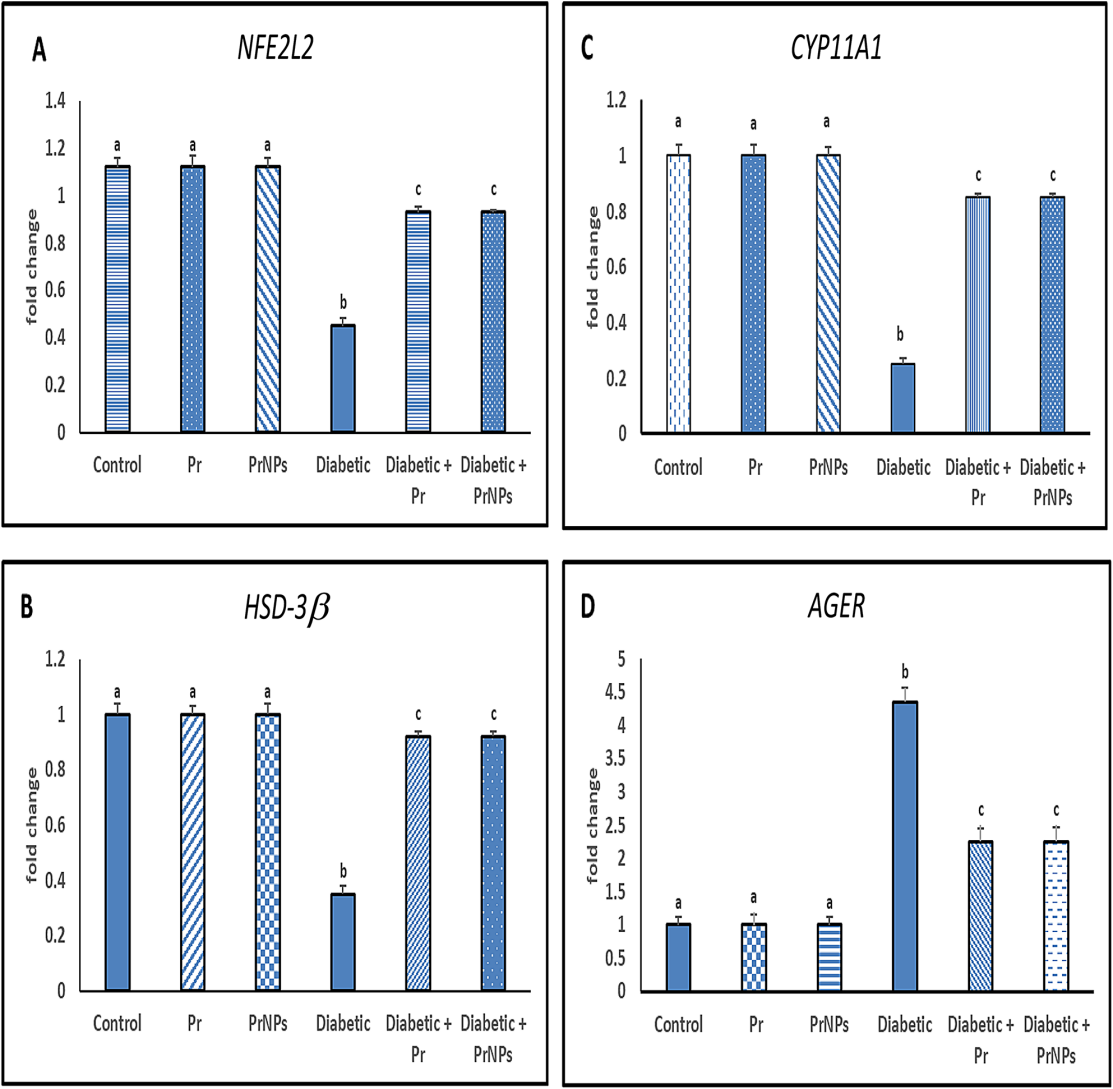
Relative gene expression analysis in different experimental groups. Each value is expressed as Mean ± standard error (*n* = 6 rats/group). Means having different letters are significantly different at (*P* ≤ 0.05).

Figure 6 revealed a significant downregulation in relative transcript levels of cytochrome P450 family 11 subfamily A member 1 (*CYP11A1*), 3β-Hydroxysteroid dehydrogenase (*HSD-3β*), and nuclear factor (erythroid-derived 2)-like 2 (*NFE2L2*), as well as the advanced glycation end-product receptor *(AGER)* gene expression, was significantly upregulated in the testis of the diabetic group relative to the control group (*p* ≤ 0.05). Treatment with Pr and PrNPs resulted in a significant upregulation in testicular mRNA expression levels of *CYP11A1*,* HSD-3β*, and *NFE2L2*, along with a significant downregulation in *AGER* gene expression in contrast to the diabetic rats (*p* ≤ 0.05). Interestingly, the diabetic group treated with PrNPs showed a significant upregulation in the expression levels of *CYP11A1*,* HSD-3β*, and *NFE2L2* genes compared to the group treated with Pr only (*p* ≤ 0.05).

### Histopathological investigation

**Fig. 7:**
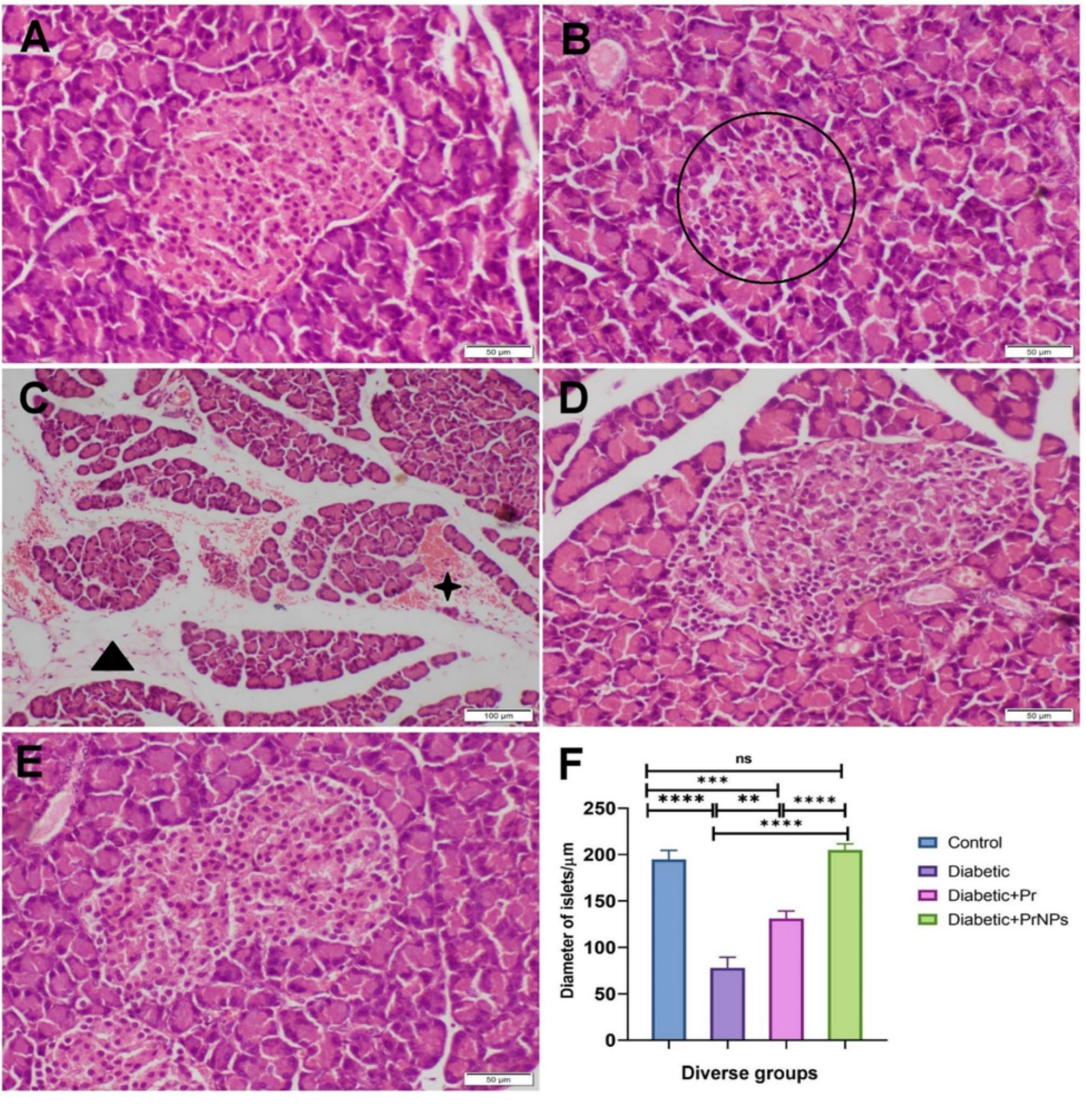
Photomicrographs representing H&E-stained pancreatic sections of diverse groups. (**A**) Control group noticed typical histological structure. (**B**,**C**) STZ group showed a remarkable reduction of islets (circle) with interstitial hemorrhage (star) and replacement of acinar cells with fat (triangle). (**D**) Diabetic rats treated with propolis, and (**E**) Diabetic rat treated with nano-propolis, both showed the normal microscopic appearance of pancreatic islets and acini. (**F**) Bar chart representing the diameter of islets in diverse groups. Data is expressed as mean ± SEM, a significant difference between groups was detected at *P* < 0.01 (**), *P* < 0.001 (***), *P* < 0.0001 (****), while (ns) means nonsignificant difference.

The pancreases obtained from the control group and those receiving propolis or nano-compacted propolis showed typical histological architecture of well-defined islets compacted with numerous beta cells (Fig. 7A). Otherwise, the diabetic group showed various histological changes. Some islets appeared atrophied whereas others demonstrated extensive degeneration of beta cells (Fig. 7B). Extensive loss of acini which was replaced by adipose tissue along with peri-acinar hemorrhage was the prominent lesion noticed in the STZ group (Fig. 7C). Regarding the propolis treated group, a significant increase in the diameter of islets and beta cell mass were noticed compared with STZ, but still diminished in comparison with the control one (Fig. 7D). Whereas a significant improvement was recorded in nano-propolis treated group and the pancreas appeared with normal histology (Fig. 7E). The morphometric analysis of pancreas reported a significant decrease in islets diameter in diabetic group compared with those of the control group. Otherwise, the propolis-treated group either crude extract or nanoemulsion displayed a significant reduction in islets diameter compared with the diabetic group, but the best improvement was noticed in the nano-propolis-treated group (Fig. 7F).

**Fig. 8:**
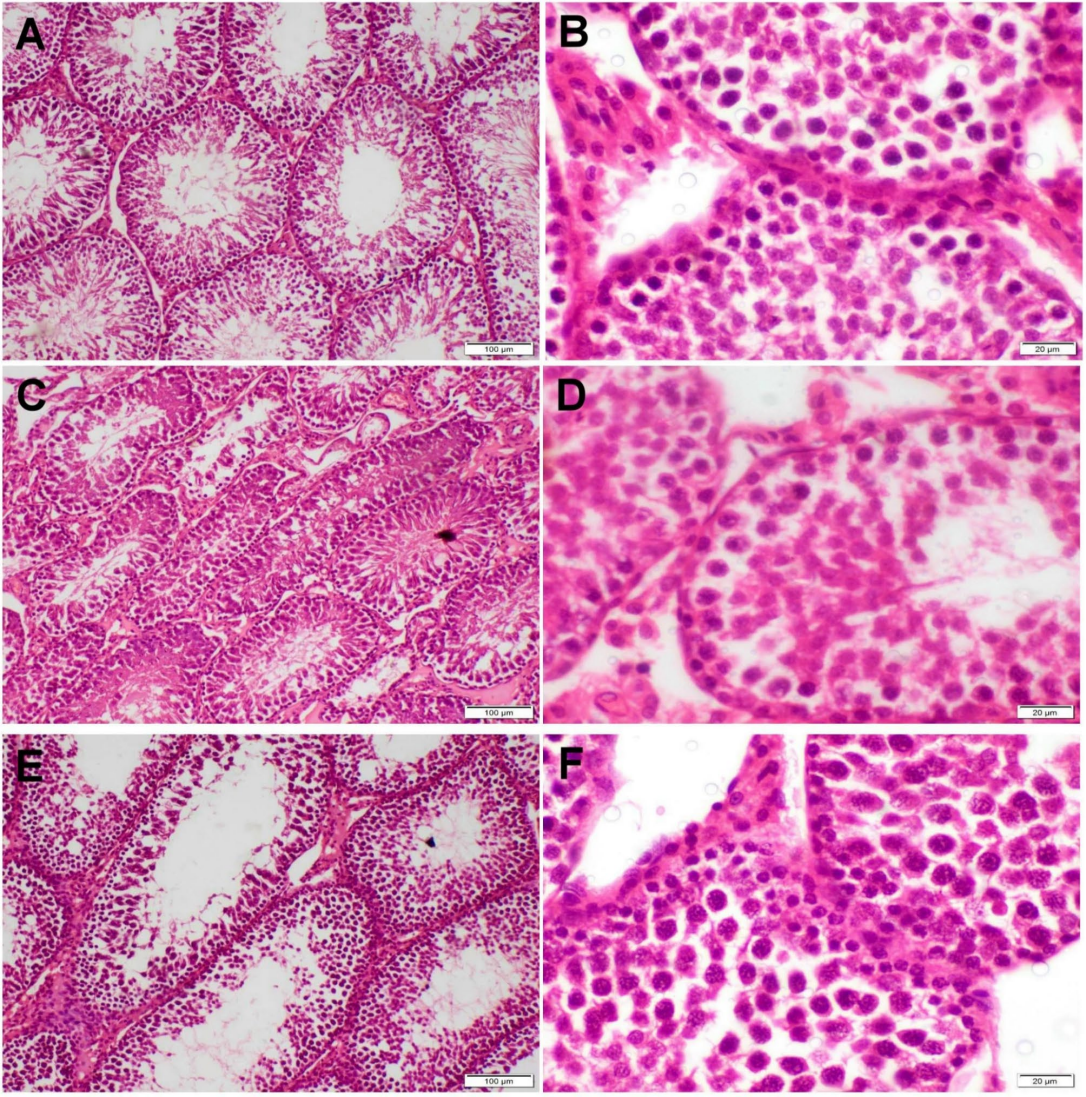
Photomicrographs representing H&E-stained testicular tissue sections of the control groups. (**A**,**B**) control negative groups, (**C**,**D**) control propolis group, and (**E**,**F**) control nano-propolis group. All these groups displayed typical testicular structures.

**Fig. 9:**
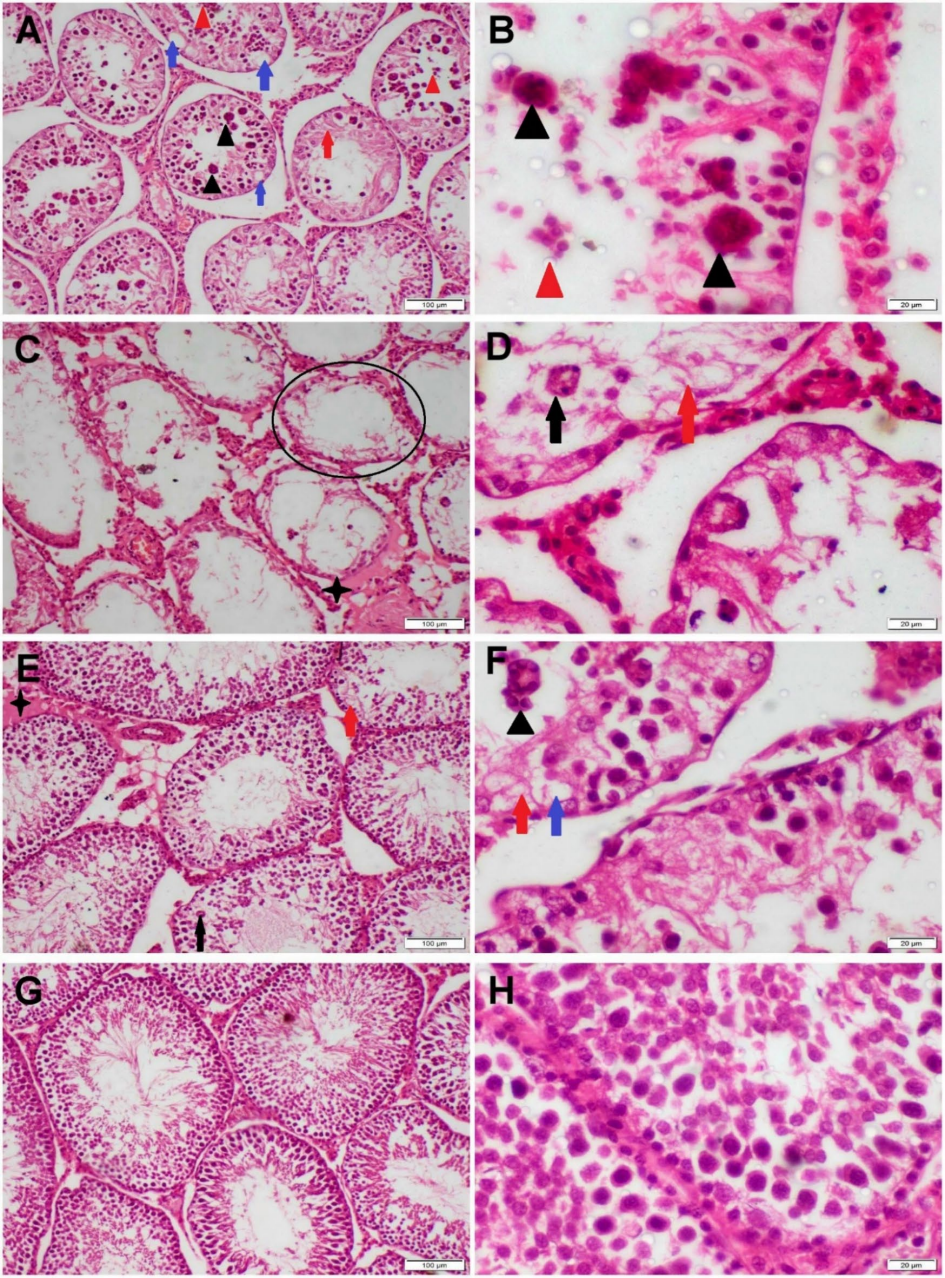
Photomicrographs representing H&E-stained testicular tissue sections of different diabetic and treated groups. (**A**–**D**) Diabetic rats showed huge spermatid giant cells (black triangle), degenerated germ cells (black arrow), desquamated germ cells (red triangle), necrotic germ cells (red arrow), vacuolation (blue arrow), interstitial edema (star), and empty seminiferous tubules (circle). (**E**,**F**) Diabetic rats treated with propolis showed moderate vacuolation (blue arrow) and necrosis of germ cells (red arrows) with interstitial edema (star). (**G**,**H**) Diabetic rats treated with nano-propolis displayed normal histological structure.

**Fig. 10:**
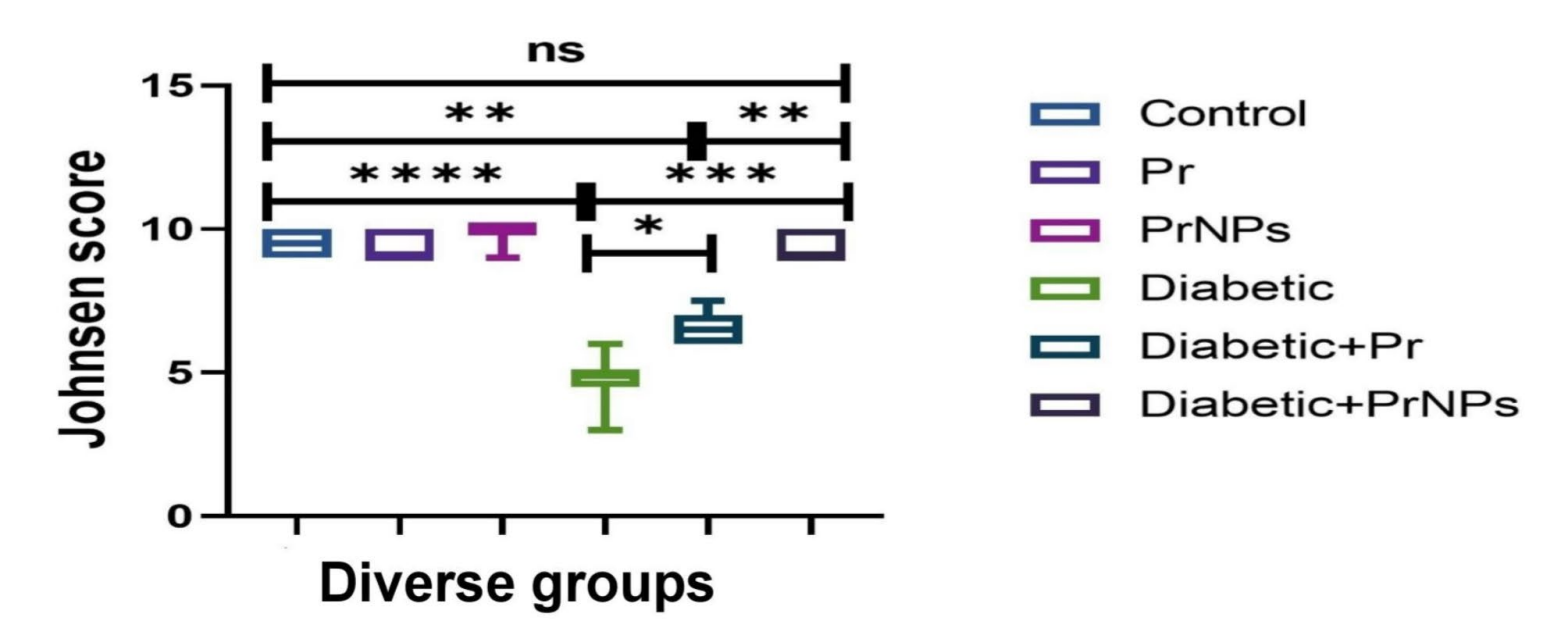
Boxplot representing Johnsen score of diverse groups. Data is expressed as min and max with line at median, a significant difference between groups was detected at *P* < 0.05 (*), *P* < 0.01 (**), *P* < 0.001 (***), *P* < 0.0001 (****), whereas (ns) means nonsignificant difference.

The testicular tissue of all control groups demonstrated normal histological structure (Fig. 8). On the other hand, diabetic rats showed severe histopathological alterations in all sections. Marked reduction of spermatogenic cell count with numerous multinucleated spermatid giant cells was the prominent lesion noticed in most sections (Fig. 9A,B). Most ST appeared empty or lined by vacuolated cells (Fig. 9C,D). Some sections showed tortuous ST along with extensive widening of interstitial tissue. Moreover, interstitial and tubular edema were other lesions observed with interstitial inflammatory cell infiltration. Regarding the treated diabetic groups, testicular sections of propolis treated group showed mild to moderate degeneration and desquamation of germinal epithelium (Fig. 9E,F), whereas those of the nano-propolis group displayed typical histological structure (Fig. 9G,H). The findings of Johnsen scoring showed a higher score in the nano-propolis-treated group as the same as the control groups. The lowest score was recorded in the STZ receiving group. Furthermore, a significant increase in this score was observed in the propolis group compared with the STZ group (Fig. 10).

## Discussion

Type 1 diabetes is a metabolic disorder characterized by hyperglycemia due to insufficient insulin production or a complete lack of insulin. It is experimentally induced by the destruction of pancreatic beta cells, typically through the administration of streptozotocin (STZ)^46,47^. The administration of STZ led to the destruction of beta cells through its oxidative stress effects^48^. In the current study, pancreatic oxidative stress was manifested by significant elevation of pancreatic MDA, NO, and depletion of GSH in the diabetic group in contrast to the control group. These findings were confirmed by histopathological investigations showing that some islets appeared atrophied, while others demonstrated extensive degeneration of beta cells. Additionally, there was a significant loss of acini, which was replaced by adipose tissue, along with peri-acinar hemorrhage, noted as the prominent lesion in the STZ group. Moreover, a notable rise in blood glucose levels was observed in diabetic rats compared to the control rats.

Diabetes impacts various bodily systems, including reproductive functions, and has been associated with male infertility due to factors like oxidative stress and hormonal imbalances^49^. The data presented in this study showed a significant elevation of testicular NO and MDA levels along with a significant reduction in their GSH content in the diabetic rats relative to the control rats. These results align with earlier researches^50–52^. NO is primarily recognized as a signaling molecule rather than a free radical, it may contribute to the generation of reactive nitrogen species, particularly peroxynitrite (ONOO−), which possesses free radical properties^53^. These reactive species have the potential to induce testicular oxidative stress and are also implicated in the development of pancreatic β-cell dysfunction. In diabetes, the overproduction of NO, often associated with increased advanced glycation end products (AGE) production results in the upregulation of nitric oxide synthase (NOS) enzymes that can facilitate the formation of reactive nitrogen species^54^.

Glutathione serves as a crucial antioxidant molecule in various tissues, including the testis and pancreas^55^. Its primary function is to shield cells from the detrimental effects of oxidative stress^56^. Our findings came in line with what was reported previously^57,58^. Moreover, in diabetes, elevated oxidative stress levels can result in the depletion of reduced glutathione (GSH)^59^. We hypothesized that the testicular GSH depletion is due to the excessive demand for GSH to neutralize ROS, diminishing the cell’s ability to combat oxidative damage effectively and rendering these cells susceptible to oxidative stress-induced damage^60^.

Lipid peroxidation products such as MDA are formed when free radicals are not adequately neutralized. These compounds could be pivotal in the onset of male infertility issues linked to diabetes mellitus^61^. Elevated MDA levels are also significant in pancreatic damage associated with diabetes. Furthermore, the excessive production of the free radical NO, influenced by STZ, is suggested to contribute significantly to the deterioration of β-cells during the early stages of DM^62^. Furthermore, the results of the current study demonstrated a significant upregulation in the relative expression of *AGERs* in the diabetic rats compared to the control rats. AGEs are pivotal in the evolution of diabetic complications involving male fertility as consistent with prior research^63^. In diabetes, the formation of AGEs occurs at an accelerated rate, with the potential for up to a 14-fold increase^64^. The upregulation of *AGER* expression can be attributed to the increased blood glucose levels that trigger AGE formation, as observed in previous studies^64,65^. Elevated levels of reducing sugars in diabetes result in the increased intracellular accumulation of glyoxal (GO), methylglyoxal (MGO), and 3-deoxyglucosone (3-DG)^66^. This surge in AGE formation leads to an upregulation of *AGER* and the subsequent generation of ROS. Upon AGE binding with AGER, this interaction prompts NADPH-oxidase activity, initiating the *Nf-κB* pathway and ultimately resulting in increased *iNOS* expression and the production of ONOO − leads to oxidative stress-induced tissue damage, including in the testes^67^. Furthermore, the results of the current study revealed significant downregulation in the relative gene expression of testicular nuclear factor, erythroid 2-like 2 (*NFE2L2*) in the diabetic rats compared to control rats. *NFE2L2* is a transcription factor that activates the oxidative stress defense system by generating antioxidant and detoxifying enzymes to protect cells from oxidizing assaults^68^. Several studies have reported the protective effects of *NFE2L2* signaling against various stress insults^69,70^. Previous studies have focused on the involvement of *NFE2L2* in DM and its complications, either in vivo^71–73^ or in vitro^74–76^. These studies have shown that *NFE2L2* down-regulation during hyperglycemia could be an important target for treatment.

The present study found that oxidative stress-induced testicular damage impairs proper spermatogenesis. This demonstrated as a substantial decrease in sperm concentration, motility, and viability along with an increase in sperm defects in the diabetic group compared to the control group that came in consistent with earlier research findings^31,77^. Lipid peroxidation (LPO) leads to damage to the axoneme, which is essential for sperm motility in addition to increased morphological abnormalities and reduced sperm viability, ultimately inhibiting spermatogenesis and resulting in a decreased sperm count^78,79^. Our results were confirmed by a histopathological investigation revealing diabetic rats with severe testicular histopathological alterations in all sections consistent with previous studies^80^.

Data presented in the current study revealed a significant decline in serum testosterone and LH in the diabetic group when compared with the control group. Research has provided evidence of a direct link between the quality of sperm parameters, and the proper functioning of steroidogenic and spermatogenic pathways^81^. It has been suggested that there may be a potential connection between increased lipid peroxidation, oxidative damage, and the impairment of the steroidogenic pathway’s^82^. Testosterone, the primary androgen responsible for spermatogenesis in the testes, is synthesized by Leydig cells under the influence of luteinizing hormone (LH)^83^. Our findings indicate a significant decline in steroidogenic capacity and Leydig cell proliferation in the diabetic group. The reduction in plasma testosterone levels noted in the diabetic group may be attributed to the downregulation of genes involved in androgen synthesis, including *HSD-3* β and *CYP11A1* reported in the current study. These results are consistent with previous research^84^. Diabetes-induced testicular injury is associated with its ability to induce damage in spermatogonia and supporting cells, such as Sertoli and Leydig cells^4^.

Hence, the utilization of antioxidants to prevent or reduce the complications associated with reactive oxygen species (ROS) for diabetes mellitus (DM) becomes essential^10^. Antioxidants effectively prevent or repair oxidative damage to a specific molecule by providing an electron to stabilize the free radical^85^. Consuming natural products rich in antioxidants like flavonoids and phenolic acids enhances the balance between oxidants and antioxidants in the body, reducing the risk of complications related to DM^86^.

Within the realm of Functional food, Propolis is a bee glue crafted by honeybees through the amalgamation of their waxes with plant resins^87^. It exhibits antioxidant activities and possesses antibacterial, anti-inflammatory, and several biological benefits^14^. Propolis provides antioxidant benefits with over 300 components, including phenolic compounds like flavonoids^14^. In the current study, Phytochemicals studies on propolis extract have identified flavonoids and polyphenolic compounds as the primary active ingredients, suggesting that both propolis (Pr) and propolis nanoparticles (PrNPs) show significant therapeutic promise in alleviating diabetic complications, likely due to the presence of significant amounts of gallic acid, chlorogenic acid, syringic acid, and rosmarinic acid, these were consistent with the previous findings ^88–90^. These phenolic compounds demonstrate strong antidiabetic, antioxidant, and hormone-regulating properties. Previous studies on gallic acid^91–93^, chlorogenic acid^94,95^, syringic acid^96–98^, and rosmarinic acid^99–101^ highlight their antioxidant, antidiabetic, and hormone-regulating properties. These effects are primarily attributed to mechanisms such as scavenging free radicals, enhancing endogenous antioxidant enzymes, inhibiting lipid peroxidation, and chelating metal ions. Additionally, these compounds modulate pro-inflammatory cytokines, reduce oxidative stress, and influence metabolic enzymes, contributing to their therapeutic potential^40,102,103^. Also, furfural, dimethyl sulfide, palmitic acid, and nonanal, all present in propolis, are bioactive compounds with promising antioxidant, antidiabetic, and male fertility-regulating properties. Furfural helps combat oxidative stress by scavenging free radicals, protecting pancreatic β-cells, and inhibiting carbohydrate-digesting enzymes, which aid in blood sugar regulation^104^. Dimethyl sulfide (DMS) and nonanal also exhibit antioxidant activity by neutralizing free radicals^105,106^.

Propolis protects against oxidative stress by enhancing antioxidant levels and enzymatic activity, reducing lipid peroxidation, and inhibiting the formation of harmful reactive oxygen species^107^. Moreover, propolis has demonstrated protective effects on the reproductive system, improving sperm quality, testosterone levels, and testicular function^108^. Despite its health benefits, propolis’ poor bioavailability, oxidative instability, and intense biotransformation constraints have hindered its application^21^. To address these issues, nanotechnology is employed to enhance the effectiveness of propolis in treating male infertility^102^. This is achieved by improving its bioavailability, stability, and targeted delivery. To the best of our knowledge, the application of propolis nanoparticles hadn’t been evaluated yet against male fertility in diabetic rats but in a similar study^103^, it has shown beneficial effects in cisplatin-induced male infertility in rats, demonstrating greater improvements than propolis alone. So, these advancements are supposed to be beneficial for diabetic individuals, who often suffer from oxidative stress, hormonal imbalances, and DNA damage in sperm. By leveraging nanotechnology, the therapeutic potential of propolis can be maximized, offering a promising natural alternative for improving male reproductive health disturbances caused by diabetes.

Our data demonstrated that treating diabetic rats with Pr and PrNPs has improved the health of both testicular and pancreatic tissues via reduction of oxidative stress markers (MDA and NO), while simultaneously boosting antioxidant capacity, as evidenced by elevated levels of (GSH). This reduction in oxidative stress and enhanced antioxidant activity contributed to increased insulin secretion by mitigating the oxidative stress induced by STZ. This, in turn, enhanced the activity of pancreatic beta cells, resulting in decreased serum glucose levels in the treated diabetic rats in contrast to the untreated diabetic rats. Histopathological investigations supported these findings, revealing that diabetic rats treated with Pr and PrNPs showed improved microscopic appearance of pancreatic islets and acini. These results align with a previous study^109^ that revealed regeneration of the beta-cells in STZ rats after the administration of Malaysian propolis.

Additionally, the administration of Pr and PrNPs leads to an increased level of luteinizing hormone (LH) in the treated diabetic rats when compared with the untreated diabetic rats due to elevated insulin levels, which act on insulin receptors in the brain, providing energy to the hypothalamus to produce GnRH that acts on the anterior pituitary to produce LH from gonadotroph cells^110^. This effect is also attributed to the ability of propolis and propolis nanoparticles to reduce oxidative stress and enhance antioxidant activity in the hypothalamus and pituitary gland. Our results agree with^111^ who showed propolis can counteract reproductive adverse effects by restoring antioxidant enzyme activity and enhancing protein content, increasing LH levels.

In the present study, the administration of Pr and PrNPs resulted in improved antioxidant activity in treated diabetic rats relative to the untreated diabetic rats, as evidenced by the increased expression of *NFE212*, the key regulator of antioxidants. These results are consistent with a previous study^112^ that demonstrated an increase in *NFE212* expression in testicular tissue of streptozotocin-induced diabetic rats following the administration of Malaysian propolis. Also, an elevation of *NFE212* expression in H9c2 cells, a cell line derived from rat heart tissue, was exposed to H2O2 following propolis extract administration^113^. These due to propolis acts as a natural activator for *NFE212*^114^. Additionally, there was further enhancement of steroidogenesis in diabetic rats treated with Pr and PrNPs compared to the untreated diabetic rats, as indicated by the increased expression of steroidogenic genes *CYP11A1* and *HSD-3β* This leads to an increase in testosterone production. These findings align with a closer study^40^, which showed that nano-form propolis and ginseng showed a promising result in counteracting the negative consequences of dexamethasone on male reproductive health by restoring the expression levels *of CYP11A1*,* HSD-3β*,* NFE212* genes in the testicular tissue.

Furthermore, the treatment of diabetic rats with either Pr or PrNPs notably led to downregulation of the relative gene expression of the *AGER*. This resulted in an improvement in glucose metabolism and reduced oxidative stress, which likely enhanced sperm quality. These improvements were evidenced by increased sperm count, viability, motility, and testicular weight, while sperm abnormalities were reduced compared to the untreated group. This result came in line with previous findings^115^ which showed that Brazilian propolis inhibited AGE accumulation in methylglyoxal (MGO)-induced glycation stress in mice due to its strong antioxidant effects.

In the current study, PrNPs exhibited superior efficacy compared to Pr alone upon administration to diabetic rats, this could be attributed to several factors. Firstly, nanoparticles offer a larger surface area, facilitating more efficient interactions with biological systems and enhancing the absorption and bioavailability of propolis’s active compounds. Moreover, they can be tailored to target specific tissues or cells through surface modifications, delivering a concentrated dose of the therapeutic agent, thus boosting its efficacy.

These conclusions were supported by histopathological examination, which revealed a significant decline and necrosis of testicular and pancreatic tissue in the untreated diabetic group compared to controls, consistent with previous findings^116^.

## Conclusion

The current study demonstrates that both propolis extract (Pr) and propolis extract nanoparticles (PrNPs) exhibit a promising therapeutic potential in ameliorating diabetic complications through their antidiabetic, antioxidant, and steroidogenesis modulatory roles. PrNPs show superior efficacy compared to Pr alone, attributed to their larger surface area, targeted delivery, protection of active compounds, and potential for reduced off-target effects. However, there is limited research on the effects of propolis, particularly regarding male fertility in the context of diabetes. Also, it is the first time to evaluate the effect of PrNPs versus male fertility in diabetic rats. This gap in literature prompted the current investigation. Further studies, including different dosage schedules, are needed for the full understanding of the potential advantages of PrNPs on fertility in diabetic conditions.

## Data Availability

Data is provided within the manuscript.

## Abbreviations

AGEs: Advanced glycation end products
CYPs: Cytochrome P450
DM: Diabetes mellitus
HSDs: Hydroxysteroid dehydrogenases
LH: Luteinizing hormone
MDA: Malondialdehyde
NO: Nitric oxide
NFE212: Nuclear factor erythroid 2-related factor 2
Pr: Propolis extract
PrNPs: Propolis extract nanoparticles
GSH: Reduced glutathione
